# Cartas al editor

**Published:** 2020-06-30

**Authors:** César Merino-Soto

**Affiliations:** 1 Universidad de San Martín de Porres Universidad de San Martín de Porres Universidad de San Martín de Porres Peru

Lima, 2 de junio de 2020

Señores Comité Editorial

Revista *Biomédica*

Bogotá, D.C

Estimados señores:

En un reciente e importante estudio aparecido en *Biomédica*^1^, se utilizó una de las escalas de percepción del estrés (*Perceived Stress Scale*) ^2^ más representativas internacionalmente para su medición cuantitativa. Aunque la amplia difusión de dicha escala pareciera contradecirlo, su estructura dimensional ha sido objeto de controversia frente a la pregunta de si sus puntajes implican una o dos dimensiones.

Los autores reportan tangencialmente que en su estudio hallaron una sola dimensión, sin más respaldo empírico que la magnitud del valor propio (*Eigenvalue*) y el porcentaje de varianza retenida por este único factor. Este resultado es afortunado porque en sus principales estudios, el autor de la escala lo usa como una medida unidimensional ^3^^-^^5^, concepción que queda explícita desde el título mismo de su primer artículo sobre la elaboración de la escala, pues lo califica como una medida “global” del estrés ^2^, y no como una medida multidimensional. Esto contrasta con el uso internacional de la escala. Efectivamente, en la mayoría de los estudios se la emplea como una medida de dos dimensiones. En la revisión sistemática de Lee ^6^ sobre estudios hasta el 2012, se señalaba como el resultado empírico más frecuente sobre la dimensión de la escala el de las dos dimensiones, ambas asociadas con el fraseo de los ítems (positivos y negativos).

Para contextualizar los hallazgos de esa revisión sistemática ^6^, en el cuadro 1 se presenta un resumen de algunas de las características más relevantes de los estudios de validación de la estructura interna de la escala en participantes colombianos ^7^^-^^9^ considerando el enfoque analítico (exploratorio o confirmatorio), el método de factorización (función de ajuste, rotación), el tamaño muestral, la versión utilizada, la consistencia interna (alfa), el número de factores empíricos obtenidos, la nominación de los factores, y la observación.

**Cuadro 1:** Revisión de estudios psicométricos colombianos

	Enfoque analítico	Método	N	R interfactorial	Versión	Alfa	Número de factores	Nominación	Observación
Campo-Arias, *et al*. (2009)	Exploratorio	Función: ML	175	0,649	PSS-10	PSS14 total: 0.87	PSS-10:	F1: percepción del estrés	No se recomienda usar puntaje total
					PSS-14	PSS10 total: 0.86	1 factor	F2: afrontamiento de los estresores	
							2 factores		
Campo-Arias, *et al*. (2015)	Exploratorio	Función: ML	366	-0,224	PSS-10	PSS14 total: 0.65	PSS14:	F1: estrés general	Sin claridad en la conclusión
		Rotación: Promax			PSS-4	F1: 0.82	2 factore	F: capacidad de afrontamiento	
Días, *et al*. (2015)	Exploratorio	Función: PCA	352	No reportado	PSS-14	PSS14 total: 0.84	PSS-14:	No reportado	Se utilizó puntaje total
						F1: 0.821	2 factores		
						F2: 0.814			

PSS: *Perceived Stress Scale*

En este sentido, hasta el momento de la elaboración de esta carta, el único enfoque analítico había sido el exploratorio, el tamaño muestral era de menos de 500, los factores se nominaban de forma diferente, la función del ajuste estadístico se utilizaba sin considerar la naturaleza categórica de los ítems ni su sensibilidad frente al incumplimiento de la normalidad multivariada, había una alta variabilidad de la correlación interfactorial e inconsistencia en su reporte, así como en el uso de un puntaje total frente al hallazgo empírico de dos factores obtenidos en la propia muestra de estudio. Este conjunto de características parece no corresponder con las actuales recomendaciones del uso de métodos factoriales exploratorios ^10^. Es evidente que es necesario incluir otros modelos, como el método *bifactor*^11^^,^^12^, el cual se viene aplicando en los estudios posteriores al 2012 ^13^^,^^14^, y con el que se ha logrado determinar consistentemente una sola dimensión de la escala. En resumen, parece que al evaluar las dimensiones de esta escala, se obtienen diferentes conclusiones según la metodología empleada.

En este contexto, se hace un llamado para que los autores expongan con mayor detalle los resultados factoriales que permiten la interpretación unidimensional (no multidimensional) de esta escala.
