# Supplementary material for: Cartas al editor
Source: Biomedica. 2020 Jun 30;40(2):415–9. [Article in Spanish] (PMC7505516)
Supplement: Related texts

# Reply: Cartas al editor

**Authors:** Deivis Nicolás Guzmán-Tordecilla, Andrés Ignacio Vecino-Ortiz, Diego Lucumí, Graciela Mentz

**Affiliations:** 1 Escuela de Gobierno, Universidad de los Andes, Bogotá, D.C., Colombia Universidad de los Andes Escuela de Gobierno Universidad de los Andes BogotáD.C Colombia; 2 Escuela de Gobierno, Universidad de los Andes, Bogotá, D.C., Colombia; Instituto de Salud Pública, Pontificia Universidad Javeriana, Bogotá, D.C., Colombia Pontificia Universidad Javeriana Escuela de Gobierno Universidad de los Andes BogotáD.C Colombia; 3 Escuela de Gobierno, Universidad de los Andes, Bogotá, D.C., Colombia Universidad de los Andes Escuela de Gobierno Universidad de los Andes BogotáD.C Colombia; 4 Department of Health Behavior and Health Education, University of Michigan, Michigan, Estados Unidos Department of Health Behavior and Health Education University of Michigan Michigan Estados Unidos

Bogotá, 2 de junio de 2020

Señores Comité Editorial

Revista *Biomédica*

La ciudad

Estimados señores;

Con relación a la carta titulada “Escala de estrés percibido (PSS) en Colombia: comentarios al estudio de Guzmán, *et al.”* (2020), agradecemos al doctor Merino-Soto por compartir sus consideraciones en torno a nuestro trabajo. Sobre sus observaciones, quisiéramos comentar lo siguiente.

Tal cual como lo menciona el Dr. Merino-Soto, actualmente no hay consenso en la literatura especializada sobre si la escala de estrés percibido (*Perceived Stress Scale*, PSS) desarrollada por Cohen, *et al.*^1^, tiene una o dos dimensiones ^2^^,^^3^. En algunos estudios, como la revisión de Lee, se señala que tiene dos dimensiones ^4^. Sin embargo, en nuestro estudio y en otros con muestras superiores a 500 observaciones, se ha encontrado una dimensión utilizando técnicas exploratorias y confirmatorias ^5^^-^^10^.

Algunos autores han sugerido que cualquier diferencia entre los factores es irrelevante y solo refleja la estructura de las oraciones de la escala ^10^. De hecho, en algunos estudios se han determinado dos dimensiones, pero sugieren usarla como una puntuación resumida ^8^^,^^11^. Por ejemplo, Reis, *et al.,* mencionan que el análisis de confiabilidad y la evidencia de la validez del constructo no proporcionaron respaldo para el empleo de dos escalas separadas en su población de estudio ^8^. Por su parte Wu, *et al.*, empleando un diseño aleatorio-longitudinal y un modelo *bifactor,* concluyeron que sus resultados proporcionaban evidencia suficiente sobre la naturaleza unidimensional de todas las formas de la PSS y respaldaron el uso de la puntuación resumida ^11^. En un estudio publicado recientemente se usaron diferentes modelos de análisis: de un factor, de dos factores correlacionados, de tres factores correlacionados y el *bifactor*, y sus hallazgos respaldaron los resultados de Mu, *et al.* sobre la naturaleza unidimensional de la PSS ^12^.

En el contexto colombiano tampoco hay un consenso frente a si la escala tiene una o dos dimensiones. En uno de sus estudios, Campo-Arias, *et al.*, establecieron que la escala tenía dos dimensiones, pero en otro de este mismo autor, se encontró una sola dimensión ^5^^,^^13^. Una de las conclusiones frente a estos hallazgos fue que las divergencias en las dimensiones observadas probablemente guarden relación con los criterios empleados para su selección ^13^. Otra explicación se relaciona con el hecho de que la consistencia interna y la solución factorial de una escala varían de una población a otra ^5^.

En este sentido, en el estudio de Lee se encontró que los profesores, los estudiantes y los trabajadores universitarios son la población en la que frecuentemente se ha aplicado la PSS ^4^, es decir que la medición del estrés con esta escala se ha limitado a grupos y condiciones específicas, por lo que la generalización de sus resultados en otros públicos es limitada ^8^.

Es posible afirmar, entonces, que no se ha podido establecer un consenso sobre las dimensiones de la PSS y que, a pesar de sus limitaciones, nuestro estudio aporta a dicha discusión. Es necesario, sin embargo, centrarse en la evaluación de las dimensiones de la PSS con diferentes métodos y grupos poblacionales en futuros estudios.

## References

[1] Cohen S, Kamarck T, Mermelstein R (1983). A global measure of perceived stress. J Health Soc Behav.

[2] Machado W, Damásio BF, Borsa JC, da Silva JP (2014). Dimensionalidade da escala de estresse percebido (Perceived Stress Scale, PSS-10) em uma amostra de professores. Psicol Reflex e Crit.

[3] Yokokura AV, Silva AA, Fernandes JK, Del-Ben CM, Figueiredo FP, Barbieri MA (2017). Perceived Stress Scale: Confirmatory factor analysis of the PSS14 and PSS10 versions in two samples of pregnant women from the BRISA cohort. Cad Saude Publica.

[4] Lee EH (2012). Review of the psychometric evidence of the perceived stress scale. Asian Nursing Research.

[5] Campo-Arias A, Bustos-Leiton G, Romero-Chaparro A (2009). Consistencia interna y dimensionalidad de la Escala de Estrés Percibido (EEP-10 y EEP-14) en una muestra de universitarias de Bogotá , Colombia. Aquichan.

[6] Luft C, de Oliveira Sanches S, Mazo GZ, Andrade A (2007). Versão brasileira da Escala de Estresse Percebido: tradução e validação para idosos. Rev Saude Publica.

[7] Mitchell AM, Crane PA, Kim Y (2008). Perceived stress in survivors of suicide: Psychometric properties of the perceived stress scale. Res Nurs Health.

[8] Reis R, Ferreira Hino AA, Romélio Rodriguez Añez C (2010). Perceived stress scale: Reliability and validity study in Brazil. J Health Psychol.

[9] Guzmán-Tordecilla DN, Vecino-Ortiz AI, Lucumí D, Mentz G (2020). El estrés crónico como mediador de la relación entre la posición socioeconómica y el cumplimiento del tratamiento farmacológico en pacientes hipertensos.

[10] Cohen S, Williamsom GM (1988). Perceived stress in a probability sample of United States. In: Spacapan S, Oskamp S, (Eds.). The social psychology of health: Claremont Symposium on applied social psychology.

[11] Wu SM, Amtmann D (2013). Psychometric evaluation of the Perceived Stress Scale in multiple sclerosis. International.

[12] Denovan A, Dagnall N, Dhingra K, Grogan S (2019). Evaluating the Perceived Stress Scale among UK university students: Implications for stress measurement and management. Stud High Educ.

[13] Campo-Arias A, Oviedo HC, Herazo E (2014). The psychometric performance of the perceived stress scale-10 on medical students from Bucaramanga, Colombia. Revista de la Facultad de Medicina.
